# Concurrent Evaluation of Mortality and Behavioral Responses: A Fast and Efficient Testing Approach for High-Throughput Chemical Hazard Identification

**DOI:** 10.3389/ftox.2021.670496

**Published:** 2021-06-15

**Authors:** Preethi Thunga, Lisa Truong, Robyn L. Tanguay, David M. Reif

**Affiliations:** ^1^Department of Biological Sciences, Bioinformatics Research Center, North Carolina State University, Raleigh, NC, United States; ^2^Sinnhuber Aquatic Research Laboratory, Department of Environmental and Molecular Toxicology, Oregon State University, Corvallis, OR, United States

**Keywords:** zebrafish behavior, high-throughput screen, zebrafish neurotoxicity, chemical hazard assessment, hazard identification, *in vivo* screening

## Abstract

The continual introduction of new chemicals into the market necessitates fast, efficient testing strategies for evaluating their toxicity. Ideally, these high-throughput screening (HTS) methods should capture the entirety of biological complexity while minimizing reliance on expensive resources that are required to assess diverse phenotypic endpoints. In recent years, the zebrafish (*Danio rerio*) has become a preferred vertebrate model to conduct rapid *in vivo* toxicity tests. Previously, using HTS data on 1060 chemicals tested as part of the ToxCast program, we showed that early, 24 h post-fertilization (hpf), behavioral responses of zebrafish embryos are predictive of later, 120 h post-fertilization, adverse developmental endpoints—indicating that embryonic behavior is a useful endpoint related to observable morphological effects. Here, our goal was to assess the contributions (i.e., information gain) from multiple phenotypic data streams and propose a framework for efficient identification of chemical hazards. We systematically swept through analysis parameters for data on 24 hpf behavior, 120 hpf behavior, and 120 hpf morphology to optimize settings for each of these assays. We evaluated the concordance of data from behavioral assays with that from morphology. We found that combining information from behavioral and mortality assessments captures early signals of potential chemical hazards, obviating the need to evaluate a comprehensive suite of morphological endpoints in initial screens for toxicity. We have demonstrated that such a screening strategy is useful for detecting compounds that elicit adverse morphological responses, in addition to identifying hazardous compounds that do not disrupt the underlying morphology. The application of this design for rapid preliminary toxicity screening will accelerate chemical testing and aid in prioritizing chemicals for risk assessment.

## Introduction

New chemicals are continually introduced into commerce and the environment. Numerous efforts have been made by the U.S. Environmental Protection Agency (EPA) and other regulatory agencies to assess hazards and exposures of these emerging chemicals and the huge backlog of untested chemicals (Pool and Rusch, 2014). ToxCast^TM^ and Tox21^TM^ use a wide variety of *in-vitro* (biochemical and cell-based) assays, and *in-silico* methods for testing large compound libraries for their potential toxicity (US EPA, 2015). Such *in-vitro* high-throughput screening (HTS) methods are useful because they are quick, relatively cheap and reduce the need for traditional animal-based toxicity testing. They have the potential to consider multiple cell types, assess the effects of mixtures, and test effects at several different doses. However, these approaches cannot capture all potential effect pathways and do not account for realistic exposure routes (National Research Council, 2007). Toxicity testing using zebrafish can inform adverse outcome pathways by capturing relevant biology from a broad spectrum of observable phenotypes across developmental stages. Data collected from such whole animal studies carry information that helps us in understanding the full implications of these chemicals in an organism. Several studies have shown that the morphological effects of chemical exposure in zebrafish increase in severity and frequency in a dose-dependent manner (Carvan et al., 2004; Dubińska-Magiera et al., 2016; von Hellfeld et al., 2020). However, detailed morphological evaluations can be resource-intensive, time-consuming, and expensive. This is especially true in an initial screening context, where evaluation of multiple endpoints is necessary to screen for all possible adverse outcomes. Given the large number of chemicals that need to be evaluated, the time and costs associated with generating such hazard data are significant and can be difficult to systematically implement.

Behavioral assays, on the other hand, are non-invasive, scalable, and relatively inexpensive (Levin and Cerutti, 2009). These responses are consequences of the integration of multiple levels of biological outcomes such as biochemical and physiological processes (Russell et al., 1990). Previously, using concentration-response behavioral profile data generated across 1060 ToxCast chemicals, we showed that chemicals altering behavioral responses at an early, 24 hpf time point can manifest in some combination of 18 specific developmental endpoints measured at 120 hpf. A majority of these chemicals that induced aberrant behavioral responses progressed from subtle effects detectable at lower concentrations of toxicant to clear impairment of movements at higher concentrations. Using morphological endpoints alone, the toxicity of many of these chemicals were not detected until higher concentrations, while some went unidentified, demonstrating the value of using behavioral assays in combination with other screening endpoints (Reif et al., 2016).

In this study, we systematically assess the usefulness of behavioral assays as a standalone evaluation method for HTS of chemicals, with the goal of minimizing the reliance on resource-intensive testing approaches. We investigate the effects of varying analysis parameters on the sensitivity achieved by our behavioral assays. We compare and characterize the value of information gained from different morphological endpoints and test for associations between the occurrence of adverse effects on these endpoints and behavioral responses. We implement benchmark dose (BMD) methodology to determine a point of departure and demonstrate the applicability of our behavioral data for risk assessment. Finally, we synthesize results to propose an integrated framework for efficient high-throughput screening of diverse chemical sets.

## Methods

### Data

The data used for this analysis include morphological assessments of 18 different endpoints recorded at 120 hpf, plus 24 hpf and 120 hpf behavioral response profiles of embryonic zebrafish that were generated across 1060 EPA ToxCast chemicals. Each chemical was tested at five different concentrations (ranging from 6.4 nM to 64 μM) in single embryo wells, with *n* = 32 replicates per concentration. The experimental design for photomotor response (PMR) assay and evaluation of multiple different endpoints are detailed in Reif et al. (2016) and Truong et al. (2014). Briefly, dechorionated embryos were distributed into the wells of 96-well plates and a total of 850 frames of digital video was recorded at 17 frames per second. The video recording included 30 s of background followed by a short pulse of light, then 9 s of dark before the second light stimuli was presented, and then 10 s of dark. These intervals were divided into separate activity periods for analysis, as described in section Sensitivity Analysis of Behavioral Responses of Methods.

### Approach

To demonstrate the value of information carried by the morphological endpoints, we quantified the change in entropy (explained in detail under section Value of Information of Morphological Endpoints) caused by removing data from one endpoint at a time. For the rest of this study, the chemical hit calls made using all the morphological endpoints were treated as the truth set (i.e., the set of chemicals that are known to cause adverse effects upon exposure). We utilized a statistical framework described in Reif et al. (2016) and conducted scenario analysis to probe the sensitivity of behavioral assays in detecting chemical hazards. Then, we subjected behavioral data to BMD modeling to comprehensively evaluate the response patterns before proposing an integrated framework that optimized sensitivity for chemical hazard identification. Utilizing the morphological screen as the “truth set” and tuning the behavioral assays to pick up these compounds ensures a robust, biologically relevant screen.

#### Value of Information of Morphological Endpoints

Entropy is a fundamental concept in information theory that quantifies the amount of information carried by any given random variable (Shannon, 1948). Previously, using Aggregate Entropy (AggE), an information theory-based method, we generated 10 Super Endpoints by clustering 18 distinct 120 hpf developmental endpoints (Mortality, Yolk Sac Edema (YSE), Pericardial Edema (PE), Axis, Eye, Snout, Jaw, Brain, Otic Vessel, Pectoral Fin, Somite, Caudal Fin, Pigment, Circulation, Trunk, Swim bladder, Notochord, Touch Response) based on the high mutual information shared between them.

As per (Zhang et al., 2016), the Super Endpoints are: SE1 = Mortality, SE2 = Craniofacial endpoints (Eye, Snout and Jaw), SE3 = Axis, SE4 = Edema (YSE and PE), SE5 = Upright body (Swim Bladder, Somite and Circulation), SE6 = Touch Response, SE7 = Pigment, SE8 = Brain (Brain, Otic Vesicle and Pectoral Fin), SE9 = Notochord distortion and SE10 = Trunk (Trunk and Caudal Fin). Briefly, AggE is a continuous value that summarizes endpoint responses for any given chemical, *C*, as:


$$\begin{array}{l} AggE\;=\;-\;\overset{32\;}{\underset{i\;=\;1}{\sum }}\;\overset{10}{\underset{j\;=\;1}{\sum }}\;p\left(B_{j}\;|C,\;X_{i}^{\;}\right)\;\log_{e}\;\left\{\;p\left(B_{j}\;|\;C,\;X_{i}^{\;}\right)\;\right\} \end{array}$$

Where X_*i*_ represents replicate *i* with *i* = 1, …,32 and *B*_*j*_ represents biological state (super endpoints) *j* with *j* = 1, …,10. The probability mass function is given by:


$$\begin{array}{l} p\left(B_{j\;}|C,\;X_{i}\right)=\frac{x_{ij}}{10} \end{array}$$

The threshold for AggE is a function of observed incidences over many individuals. Specifically, the concentration-wise AggE threshold is the critical value of a one-sided chi-square test with a significance level of 0.05. Any chemical with an aggregate entropy higher than the threshold is flagged as a “hit,” as it elicits responses across tested endpoints. To capture the value of information gained from each of these super endpoints, we calculated the change in AggE caused by removing each of the super endpoints, one at a time, for all chemicals. Kolmogorov–Smirnov (KS) test was used to compare the empirical cumulative distribution functions (eCDF) of the resulting aggregate entropies and identify statistically significant differences in the distribution of AggE caused by the removal of any given endpoint. This was done independently for every concentration. The level of reduction in the entropies or the extent of the shift in its overall distribution would directly correspond to the amount of information carried by that super endpoint.

#### Concordance of Behavioral Responses and Morphology Super Endpoints

In this section, we investigated the sensitivity of behavioral assays in detecting chemicals that elicited an adverse response in any one super endpoint (SE) at a time. Each of the 10 SE was used as the truth set, in turn, to evaluate performance across SEs. A chemical was considered a “hit” using a super endpoint if the incidence of malformations in that super endpoint significantly exceeded (Fisher's exact test *p*-value < 0.05) the background (control) incidence rate for the same. Methods used to consider a given chemical as a “hit” in the behavioral assays are detailed in section Sensitivity Analysis of Behavioral Responses.

#### Sensitivity Analysis of Behavioral Responses

120 hpf morphological data was analyzed using contingency tables and Fisher's exact test to check if the proportion of fish with adverse endpoints among the controls significantly differed from that of the treated samples (Reif et al., 2016). A chemical was considered a hit if it affected any endpoint(s) significantly and passed the *p*-value threshold set for the Fisher's test.

Early embryonic behavioral profiles were analyzed by partitioning them into three separate intervals: Background (basal activity observed before first light pulse), Excitatory (a single peak movement in response to first light pulse) and Refractory (period of inactivity observed after second pulse of light). Chemicals that elicited adverse behavioral responses in any of these intervals, at any concentration, constituted the list of hits called using 24 hpf behavioral data. For every concentration, the behavioral response patterns of the treated samples within each interval was compared to that of the controls using the Kolmogorov–Smirnov test (Bonferroni-corrected *p*-value threshold = 0.05 /5 concentrations = 0.01). If the percent change in activity patterns of the treated wells when compared to that of the controls exceeded hyper- or hypoactivity thresholds, it was considered a hit. Parameters used in the methods described above were tuned to achieve improved sensitivities in chemicals' detection. We systematically swept through a defined range of values for 4 main parameters: (a) the percent-change threshold for hypo activity, (b) the percent-change threshold for hyperactivity, (c) number of significant morphological endpoints used to call hits on chemicals and lastly, (d) the *p*-value threshold used for the Fisher's test to correct for testing of multiple of endpoints in morphology. The sensitivity and specificity of the behavior data to detect chemicals that elicit adverse morphological responses were recorded in each case.

#### Benchmark Dose (BMD) Modeling of Behavioral Data

We carried out quantitative dose-response analysis of behavioral data using US EPA's BMDExpress (Version 2.3) to estimate points of departure of various chemicals (Martin et al., 2009; Davis et al., 2011; Phillips et al., 2019). Each chemical set was subjected to a benchmark dose (BMD) analysis independently. For data evaluation and model selection, a general framework as described in the Benchmark Dose Modeling Technical Guidance Document (Davis et al., 2011; US EPA, 2012) was followed for every chemical set. To prevent fitting models to non-responsive data, William's Trend test was first carried out with FDR adjusted *p*-value cutoff of 0.05. A benchmark response (BMR) was defined as a 10% change relative to the background response. A low BMR such as this was chosen because we wanted the POD estimation to be lenient to reduce the number of false negatives while broadly screening for potential toxicities of chemicals. Then, BMD estimation was done by subjecting the filtered data to a parametric curve-fitting process using all non-linear continuous models available in the software. From this suite of models, a winning model was chosen for each chemical independently based on multiple criteria such as goodness of fit, the uncertainty around the BMD estimates, range of the benchmark dose estimate, and so on. Specifically, models with a goodness of fit *p*-value below the significance level (*p* < 0.1), BMD/BMDL ratio >20, and BMD estimates falling outside the minimum and maximum (0 and 64, respectively) doses were removed. From the remaining models, the one with the lowest Akaike Information Criterion (AIC) was chosen as the best model and its BMDL was used as a POD estimate. Finally, a chemical with a POD estimate within the tested range of doses was considered a hit.

#### Evaluation of Behavior and Mortality in Predicting Morphological Effects

To assess reliability of calls made using behavioral responses, the chemical hit calls made using all morphological endpoints were treated as the truth set. Then, we combined data from both 24 hpf and 120 hpf behavioral assays with mortality to assess concordance between the combined assay and morphology assessments at each concentration. F1 score was calculated as $\frac{2*PP}{2*PP\;+\;PN\;+\;NP}$ and Concordance was calculated as $\frac{\;PP\;+\;NN}{PP\;+\;NN\;+\;PN\;+\;NP}$, where PP is a hit in both morphology and the combined assay (behavior + mortality); NN represents no hit in either assay; NP represents a negative or no call in morphological assessment and hit using the combined assay, and PN is a hit in morphological assessment but not in the combined one.

## Results

### Value of Information of Morphological Endpoints

**Figure 1:**
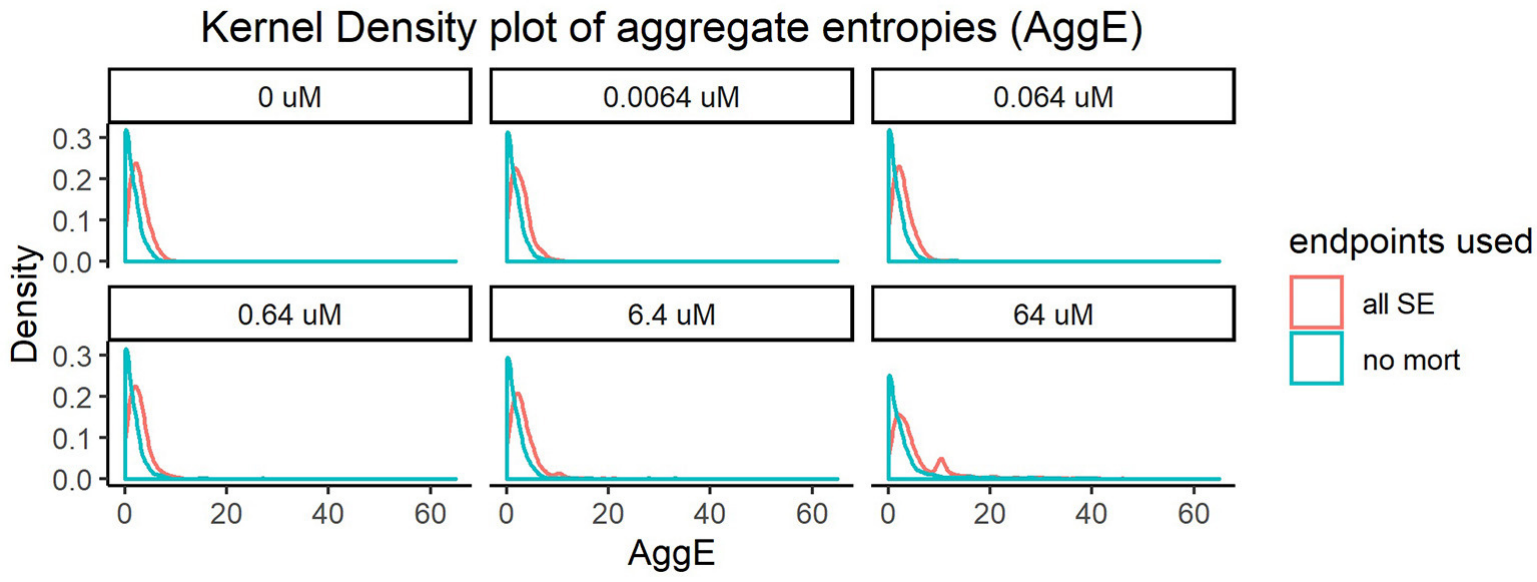
Kernel density plot of Aggregate entropies (AggE) of 1060 ToxCast chemicals before and after removing mortality information. The density histogram plots AggE on the horizontal axis. The red line represents the distribution of AggE for chemicals obtained by summarizing information from all Super Endpoints (all SE) and the blue line represents AggE of chemicals after excluding data from mortality endpoint.

Every super endpoint, upon its removal, caused a statistically significant change in the distribution of the resulting aggregate entropies in at least one concentration. Importantly, removing mortality information resulted in the highest shift in the distribution of AggE even at the lowest tested concentration (Figure 1). This indicates that this endpoint is data rich and therefore will be most suitable for quick preliminary screening of a diverse chemical set across a range of concentrations. However, this simple assay screening for just mortality alone would fail to capture chemicals that disrupt the underlying physiology but do not result in death.

The shifts in distribution resulting from removing other super endpoints, although statistically significant, were less dramatic. We hypothesized that the effects of these endpoints might appear diminished when compared with mortality, since the frequency of mortality was much higher than the frequency of occurrence of adverse effects in any other endpoint. To correct for this, we removed individuals showing mortality and re-did the analysis with the remaining super endpoints. This resulted in noticeable shifts in entropies for every super endpoint, in at least one concentration (Supplementary Figures 1–9). For instance, removing information from Edema (SE4, Supplementary Figure 3) caused statistically significant changes in distribution of AggE even in the lowest tested concentration (KS test *p*-value < 0.05). Quantifying the change in AggE in this manner highlights the importance of integrating data from a rich suite of endpoints for characterizing chemical hazards. However, screening thousands of chemicals by detailed evaluations of every super endpoint described above can be time-consuming and resource-intensive. Therefore, we need scalable assays that are representative of such diverse biological endpoints, which when coupled with mortality can maximize the sensitivity of chemical hazard identification.

### Concordance of Behavioral Responses and Morphology Super Endpoints

**Figure 2:**
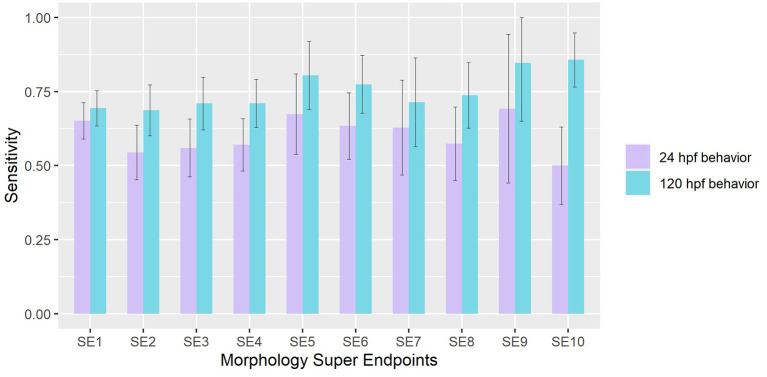
Sensitivity of 24 hpf and 120 hpf behavior in detecting chemicals that affect each morphological super endpoint. Error bars represent 95% confidence intervals.

Using approaches detailed in sections Value of Information of Morphological Endpoints and Sensitivity Analysis of Behavioral Responses under Methods, we compared the sensitivities of behavior in detecting chemicals that elicited adverse effects in each Super Endpoint (SE) separately. When mortality (SE1) was treated as the truth set, we found that both 24 hpf and 120 hpf behavioral assays have sensitivities of 0.65 and 0.7 respectively. Across all SE, we observed that 120 hpf behavior showed higher sensitivities in detecting chemicals that elicited morphological malformations when compared to 24 hpf behavior (Wilcoxon rank sum test *p-*value < 0.05) (Figure 2). The former was most sensitive in picking up hits made using super endpoints 10 (# of compounds that showed adverse effects in SE10 = 56), obtained by collapsing CFIN and TRUN, and super endpoint 9 (# of compounds that showed adverse effects in SE9 = 13) which represents Notochord distortion. These results reinforce the idea that behavior is a direct indicator of underlying physiology.

### Sensitivity Analysis of Behavioral Responses

**Figure 3:**
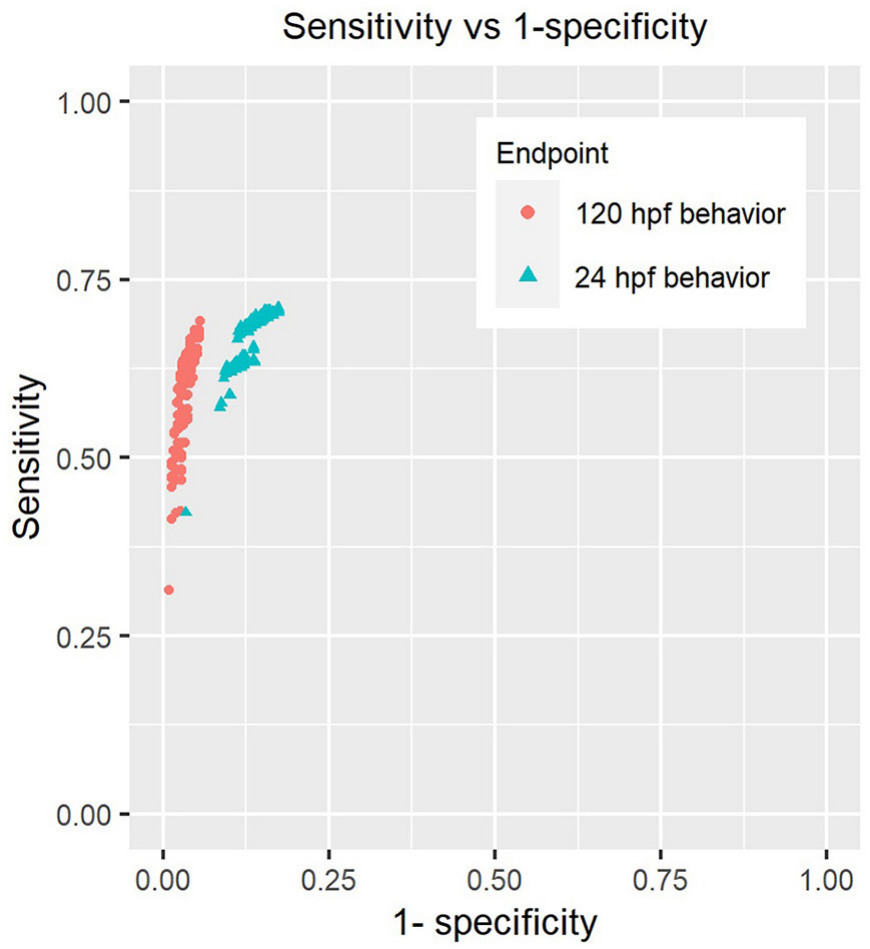
Sensitivity analysis of behavioral endpoints. Each point on the plot represents the sensitivity and specificity of these assays when using a combination of parameters described in section Sensitivity Analysis of Behavioral Responses of Methods to call hits using behavior and morphology (Parameters include the number of endpoints used to make “affected” calls in morphology, *p*-value threshold for Fisher's exact test, percent change thresholds for hypo and hyper-activity).

We tested a total of 416 different scenarios by sweeping across values ranging from 0.1 to 0.75 for hypo and hyper-activity thresholds, minimum number of adversely affected morphological endpoints varying from 1 to 10 and used significance levels for Fisher's test between 0.05 and 0.002 in order to correct for testing of multiple of endpoints. We found that the sensitivities of both 24 hpf and 120 hpf behavior were not altered drastically by the choice of parameters (Figure 3). We did not identify any combination of values for these parameters that would either inflate or diminish the sensitivity of chemicals' detection by a large extent.

Next, we compared the sensitivities of behavior in picking up chemicals with existing hazard information from EPA's CompTox Chemicals dashboard (Supplementary Table 1). These included subsets of the Phase 1 and Phase 2 ToxCast chemicals that were listed under six different categories: NeuroTox (chemicals demonstrating effects on neurodevelopment), Dev_NT (chemicals triggering developmental neurotoxicity), human neurotoxicants, Bisphenols, Eu Biocides, and Pesticides (compounds used as active ingredients in pesticides and biocides). Although 120 hpf behavior showed higher sensitivities across most categories, 24 hpf behavior consistently picked up more unique hits that were not picked up by the other two assays. This highlights the value of combining data from both behavioral assays in picking up additional information that might otherwise be missed in conventional morphological phenotypic assessments.

### Benchmark Dose Modeling of Behavioral Data

**Figure 4:**
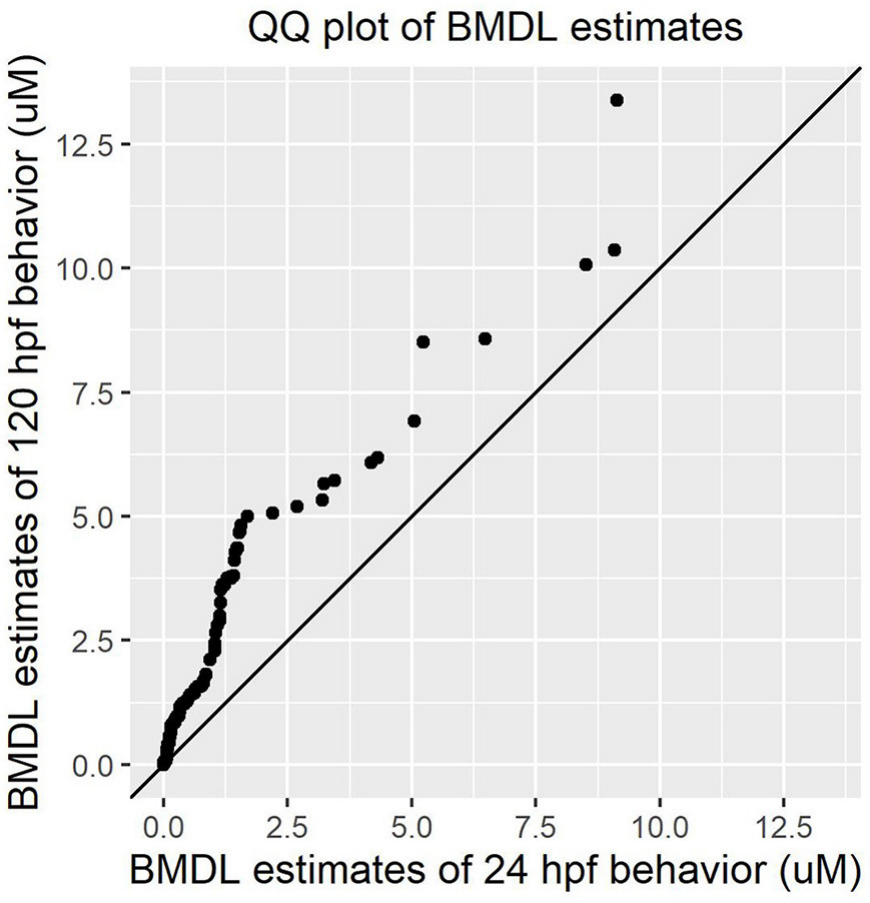
QQ plot comparing the Benchmark Dose potency estimates for 110 chemicals that were picked by both 24 hpf and 120 hpf behavioral data.

**Figure 5:**
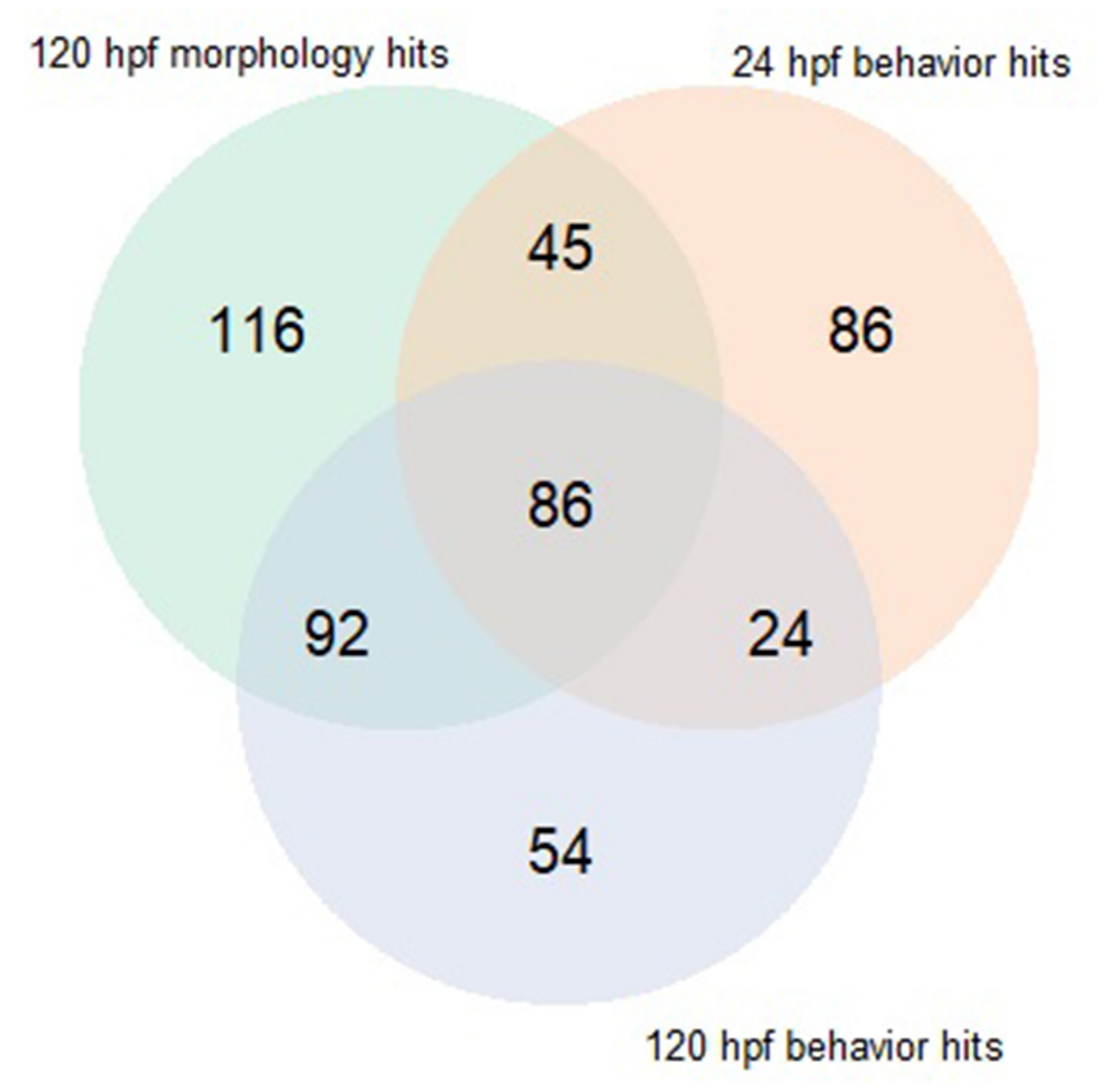
Venn diagram comparing hits made using behavioral assays when subjected to BMD modeling to those made using morphology screen.

Both early time point embryonic responses and 120 hpf movement profiles were subjected to BMD analysis independently. As we previously showed that the excitatory interval is most sensitive to chemical perturbations (Reif et al., 2016), within this interval, the distance moved was summed across all time points for every replicate and used as the continuous response input for BMD analysis. Using 24 hpf behavioral data that was available for 1060 ToxCast chemicals, we found that 278 chemicals passed the trend test indicating the presence of a dose response effect, 241 of which met all the criteria described in section Benchmark Dose (BMD) modeling of Behavioral Data under Methods. These chemicals were considered as hits. Similarly, using data from the 120 hpf assay, 256 chemical hit calls were made. One hundred and ten chemicals showed up as hits in both behavioral assays of which 86 showed adverse morphological effects. Figure 4 shows a QQ plot comparing the BMDL estimates for the 110 compounds that were picked up by both behavioral assays. The skewness in the data indicates that chemicals elicited a 10% change in responses, relative to the background, at slightly lower doses at 24 hpf when compared to 120 hpf (refer to Supplementary Table 2 for actual doses). However, this could be a consequence of different models with varying parameters being fit to the individual data sets. By integrating information from both behavioral assays, we were able to capture 223 out of the 339 (~65%) chemicals that elicit 5 dpf developmental abnormalities (Figure 5). In addition, we found 164 chemicals displaying behavioral abnormalities yet no significant alterations in morphology. However, these assays put together still do not capture the entirety of compounds resulting in adverse morphological effects. In the following section, we assess the added value of utilizing information from mortality screen together with these behavioral assays in identifying chemical hazard.

### Evaluation of Behavior and Mortality in Predicting Morphological Effects

Table 1 compares concordance of results between the combined assay (24 hpf behavioral assay, 120 hpf behavioral assay, and mortality) and a detailed morphological screen. Across all concentrations, we found that data from both behavioral responses, when combined with the hits made using mortality, comprises 83% of the chemicals that resulted in adverse morphological effects. This combined assay has a sensitivity of 0.76 (Table 1, True Positive = 279; False Negative = 60).

**Table 1:** Showing performance evaluations of the combined assay (24 hpf and 120 hpf behavior, and mortality) and detailed morphological screen.

**Concentration**	**PP**	**PN**	**NP**	**NN**	**F1 score**	**Concordance**
0.0064 uM	44	4	2	1008	0.936	0.994
0.064 uM	18	5	2	1033	0.837	0.993
0.64 uM	33	10	7	1008	0.795	0.984
6.4 uM	32	22	39	965	0.512	0.942
64 uM	161	52	106	739	0.671	0.851
Overall	279	60	88	631	0.79	0.86

*PP represents a hit in both assays; NN represents no hit in both assays; NP represents a negative or no call in morphological assessment and hit using behavior and mortality, and PN represents the opposite*.

## Discussion

Our results have demonstrated that concurrent evaluation of mortality and behavioral responses is a fast and efficient testing approach for high-throughput chemical hazard identification. Using this framework, we can achieve high sensitivity and specificity in detecting chemicals that exhibit heightened bioactivity. We have shown that behavioral assays are also useful in picking up compounds that do not otherwise elicit any obvious morphological effects, and hence might be missed in a conventional screening setup. Such chemicals manifesting only behavioral abnormalities should still be cause for additional scrutiny. For preliminary screening of a vast number of compounds, we propose that acute toxicity can be determined based on a positive outcome in any one of the three assays described in this study (24 h post-fertilization behavior, 120 h post-fertilization behavior and mortality).

Several chemicals went undetected in our morphology screen indicating the need to expand our net for chemical hazard identification (Supplementary Table 1). An ideal screening platform should be efficient with regard to time and costs, and easy to scale while still having biological relevance. From a HTS context, this combined assay reduces the need for carrying out detailed evaluations of morphology, thereby diminishing the effects of any bias or variability resulting from those evaluations and helps improve concordance across multiple labs. When study goals differ from screening, then the relative merits of detailed evaluation versus scalability in concentration range and replicates may differ in the hazard identification context discussed here, multiple concentrations and robust replicate count are critical to dose response modeling of such data to holistically evaluate patterns in behavior and identify any deviations in responses. To obtain reliable estimates of the BMD and avoid interpolation at the BMR, it is important to have studies, similar to this one, with multiple doses, several of which are near the level of the BMR. In fact, working with additional concentrations may have allowed us to capture chemicals that were otherwise missed in the current screening set up. The BMD methodology is convenient for analyzing behavioral data for many reasons. Using the BMD approach allows for a comprehensive analysis of the behavioral response profiles by paying attention to the treatment group variability while assessing model fits. We used low BMR thresholds as a basis for the analysis to increase the sensitivities of chemicals' detection because this is a screening-level assessment. Although additional careful considerations need to be carried out while using modeling to report safe levels of exposure of chemicals, here, we illustrate the usefulness of benchmark dose analysis in obtaining the preliminary point of departure estimates from behavioral studies for screening purposes. In addition, to utilize behavioral assays as a standalone assessment tool, these assays should be adjusted—by adding additional doses and, replicates per dose—to increase sensitivity while ensuring sufficient signal to background.

To summarize, we have shown that early time point behavioral responses and mortality are indicative of acute toxicity of chemicals. Previously, we have demonstrated the added value of integrating information from these behavioral assays with other *in vitro* studies in elucidating important biochemical targets (Reif et al., 2016). In addition, due the non-invasive and non-destructive nature of this screening framework, chemicals showing evidence for hazard in early life stages can even be followed up with targeted studies and/or experimental interventions in later stages of development to better understand mechanisms of toxicity. Therefore, this low-cost, sensitive hazard identification step, when combined with more sophisticated confirmatory assays using tiered approaches such as those described within the Integrated Approaches to Testing and Assessment (IATA) framework, will augment the chemical hazard characterization process and guide regulatory decisions (Tollefsen et al., 2014).

## Data Availability Statement

The data supporting the conclusions of this article will be made available by the authors upon request.

## Ethics Statement

The animal study was reviewed and approved by IACUC of Oregon State University.

## Author Contributions

PT drafted the manuscript, performed all analysis, and implemented original code. LT and RT generated experimental data. RT and DR obtained funding. All authors contributed to the study design and editing the manuscript and agreed to accountability for the content.

## Conflict of Interest

The authors declare that the research was conducted in the absence of any commercial or financial relationships that could be construed as a potential conflict of interest.
