# Supplementary material for: Concurrent Evaluation of Mortality and Behavioral Responses: A Fast and Efficient Testing Approach for High-Throughput Chemical Hazard Identification
Source: Front Toxicol. 2021 Jun 15;3:670496. doi: 10.3389/ftox.2021.670496 (PMC8915815; doi:10.3389/ftox.2021.670496)
Supplement: Supplementary file 1 [file Data_Sheet_1.docx]

Supplementary Material

| 24 hpf behavior | | | | | | 120 hpf behavior | | | | | |
| --- | --- | --- | --- | --- | --- | --- | --- | --- | --- | --- | --- |
|  | NeuroTox | Dev_NT | Bisphenol | Eu Biocides | Pesticides |  | NeuroTox | Dev_NT | Bisphenol | Eu Biocides | Pesticides |
| TP | 10 | 3 | 2 | 8 | 56 | TP | 11 | 3 | 2 | 10 | 57 |
| FP | 10 | 0 | 0 | 7 | 39 | FP | 2 | 0 | 0 | 3 | 8 |
| FN | 5 | 0 | 1 | 5 | 34 | FN | 4 | 0 | 1 | 3 | 33 |
| TN | 40 | 7 | 4 | 42 | 249 | TN | 48 | 7 | 4 | 46 | 280 |
| n | 65 | 10 | 7 | 62 | 378 | n | 65 | 10 | 7 | 62 | 378 |
| Sens | 0.667 | 1 | 0.667 | 0.615 | 0.622 | Sens | 0.733 | 1 | 0.667 | 0.769 | 0.633 |
| Spec | 0.8 | 1 | 1 | 0.85 | 0.9722 | Spec | 0.96 | 1 | 1 | 0.938 | 0.972 |

**Supplementary Table 1**. Showing sensitivity of behavioral endpoints in detecting chemicals with existing hazard information. TP represents a hit in both assays; FP represents a hit in behavioral assay alone; FN represents a hit in morphology assessment and TN represents no hit in either assay.

| BMDL_24hpf | BMDL_120hpf |
| --- | --- |
| 9.94E-38 | 2.27E-08 |
| 9.39E-13 | 6.92E-08 |
| 1.73E-11 | 1.94E-05 |
| 2.14E-11 | 0.000616 |
| 5.66E-09 | 0.00209684 |
| 9.42E-08 | 0.00487014 |
| 4.26E-07 | 0.0102272 |
| 1.52E-06 | 0.0147107 |
| 4.28E-06 | 0.0157198 |
| 1.70E-05 | 0.0209448 |
| 2.47E-05 | 0.0357516 |
| 4.16E-05 | 0.0401844 |
| 9.31E-05 | 0.0470651 |
| 0.00184248 | 0.0487146 |
| 0.0162451 | 0.0504812 |
| 0.0162777 | 0.0505764 |
| 0.029359 | 0.0602654 |
| 0.0318916 | 0.0611977 |
| 0.0325965 | 0.0661159 |
| 0.0420418 | 0.0785354 |
| 0.0434731 | 0.0806602 |
| 0.04594 | 0.119032 |
| 0.0540523 | 0.119917 |
| 0.062973 | 0.15036 |
| 0.0630577 | 0.235825 |
| 0.066406 | 0.251203 |
| 0.0731086 | 0.262447 |
| 0.0734608 | 0.313452 |
| 0.0868349 | 0.328215 |
| 0.0895561 | 0.403689 |
| 0.106209 | 0.423244 |
| 0.11596 | 0.437628 |
| 0.118206 | 0.501435 |
| 0.121074 | 0.569311 |
| 0.12909 | 0.576803 |
| 0.144697 | 0.656493 |
| 0.147001 | 0.793397 |
| 0.166455 | 0.806563 |
| 0.191722 | 0.850283 |
| 0.196798 | 0.856251 |
| 0.234697 | 0.856401 |
| 0.245937 | 0.934234 |
| 0.250456 | 0.935916 |
| 0.258232 | 0.960545 |
| 0.27587 | 0.962999 |
| 0.314405 | 0.984826 |
| 0.32142 | 1.08678 |
| 0.325234 | 1.13863 |
| 0.33184 | 1.16111 |
| 0.34757 | 1.16326 |
| 0.378328 | 1.23008 |
| 0.429167 | 1.23601 |
| 0.445973 | 1.25653 |
| 0.450339 | 1.26917 |
| 0.474795 | 1.2758 |
| 0.485568 | 1.32304 |
| 0.517309 | 1.38602 |
| 0.541002 | 1.41668 |
| 0.569642 | 1.41877 |
| 0.590304 | 1.42028 |
| 0.59343 | 1.42708 |
| 0.627471 | 1.43748 |
| 0.630289 | 1.44944 |
| 0.640696 | 1.52129 |
| 0.694193 | 1.56869 |
| 0.733408 | 1.57148 |
| 0.771574 | 1.58103 |
| 0.799694 | 1.63068 |
| 0.808474 | 1.63586 |
| 0.811275 | 1.69054 |
| 0.856852 | 1.80411 |
| 0.859018 | 1.81386 |
| 0.938822 | 2.11029 |
| 1.0276 | 2.30054 |
| 1.03059 | 2.3871 |
| 1.03852 | 2.43748 |
| 1.04274 | 2.65173 |
| 1.09017 | 2.80152 |
| 1.13625 | 2.89829 |
| 1.13911 | 3.00677 |
| 1.14854 | 3.26714 |
| 1.15578 | 3.53064 |
| 1.17925 | 3.62119 |
| 1.21312 | 3.62802 |
| 1.22366 | 3.63005 |
| 1.27155 | 3.75258 |
| 1.36085 | 3.75471 |
| 1.36801 | 3.78319 |
| 1.40553 | 3.80807 |
| 1.43378 | 4.12007 |
| 1.43485 | 4.27624 |
| 1.4807 | 4.357 |
| 1.49208 | 4.35855 |
| 1.52726 | 4.66712 |
| 1.5381 | 4.69884 |
| 1.55564 | 4.81377 |
| 1.68746 | 5.00789 |
| 2.199 | 5.07323 |
| 2.69647 | 5.18892 |
| 3.19936 | 5.32076 |
| 3.23841 | 5.64881 |
| 3.45161 | 5.71427 |
| 4.17757 | 6.08479 |
| 4.31398 | 6.1773 |
| 5.0529 | 6.91463 |
| 5.23166 | 8.50171 |
| 6.48547 | 8.58215 |
| 8.51336 | 10.0699 |
| 9.07815 | 10.3601 |
| 9.14037 | 13.3804 |

**Supplementary Table 2**. Showing BMDL values of 24 hpf behavior and 120 hpf behavior

Supplementary figures

**Supplemental Figures 1-9** Show difference in distributions of AggE when removing one Super Endpoint (SE) at a time, after correcting for mortality. In each figure, the red line represents the distribution of AggE for chemicals obtained by summarizing information from SE 2-10 and the blue line represents AggE of chemicals after excluding data from the corresponding SE as indicated within each plot.


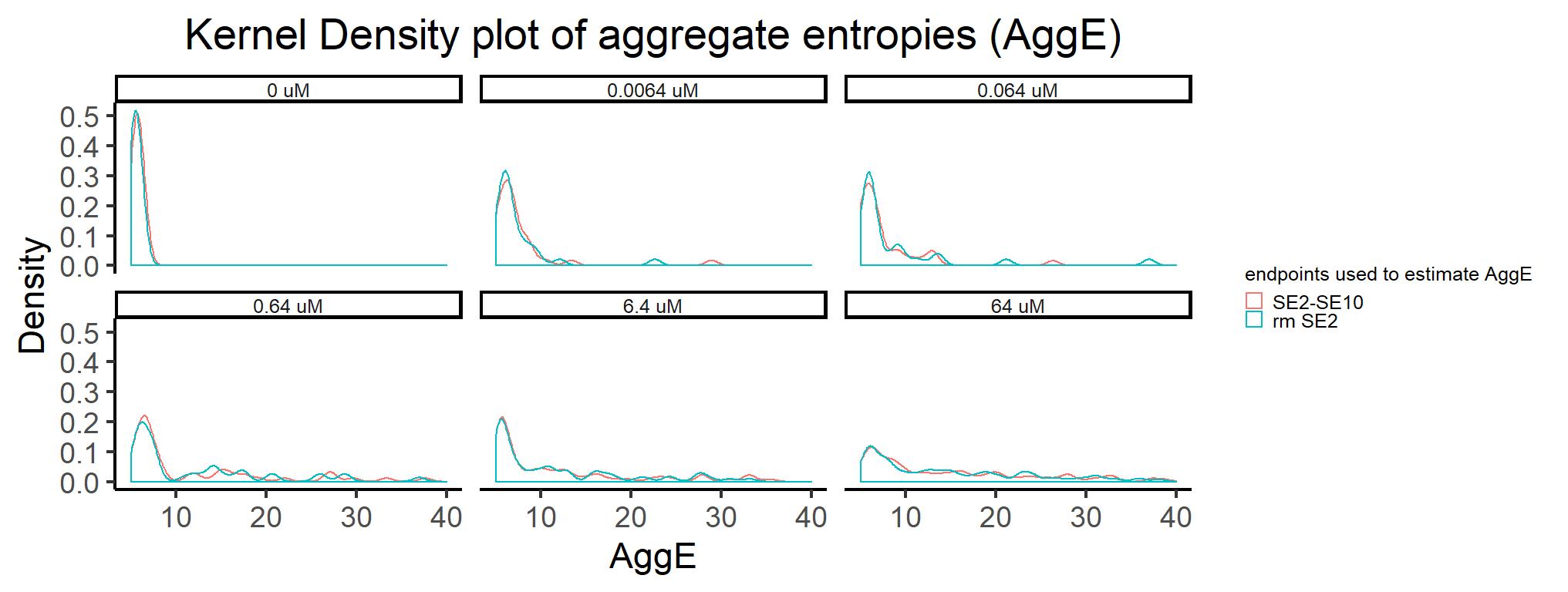


**Supplemental Figure 1** Kernel density plot of Aggregate entropies (AggE) of ToxCast chemicals before and after removing Craniofacial endpoints (SE2). The density histogram plots AggE on the horizontal axis.


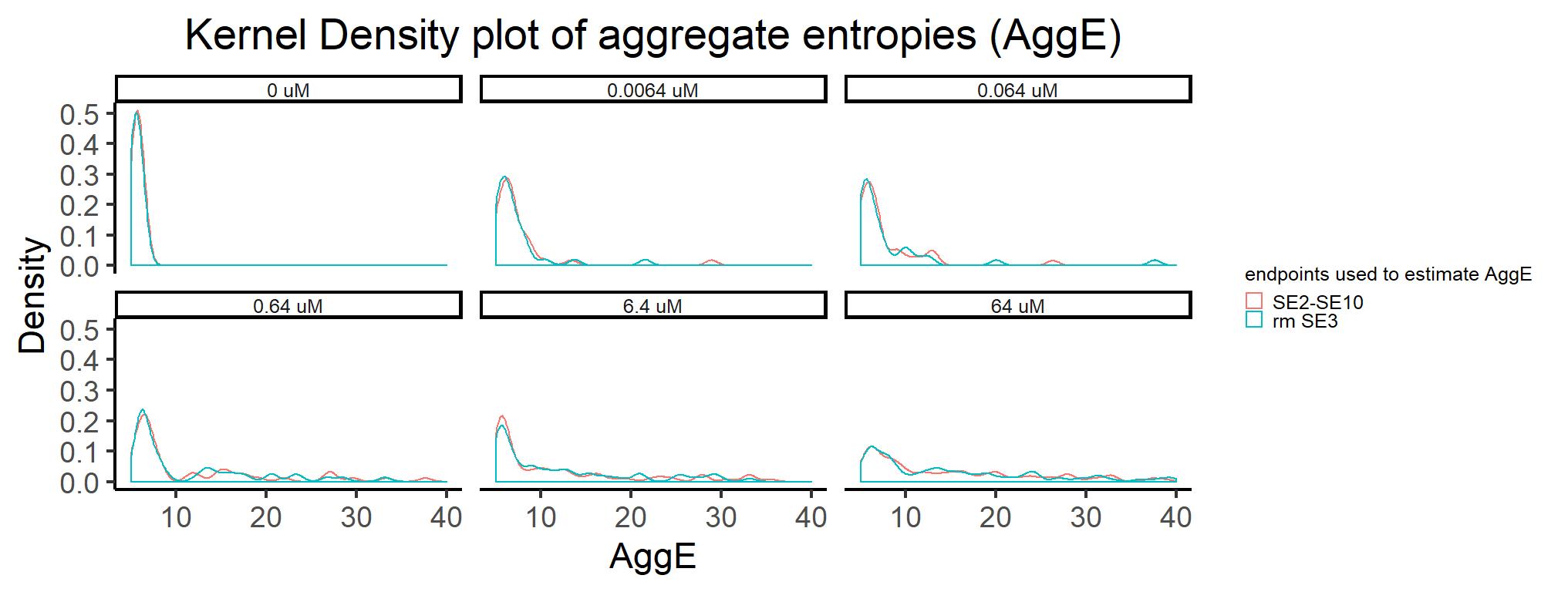


**Supplemental Figure 2** Kernel density plot of Aggregate entropies (AggE) of ToxCast chemicals before and after removing ‘Axis’ (SE3). The density histogram plots AggE on the horizontal axis.


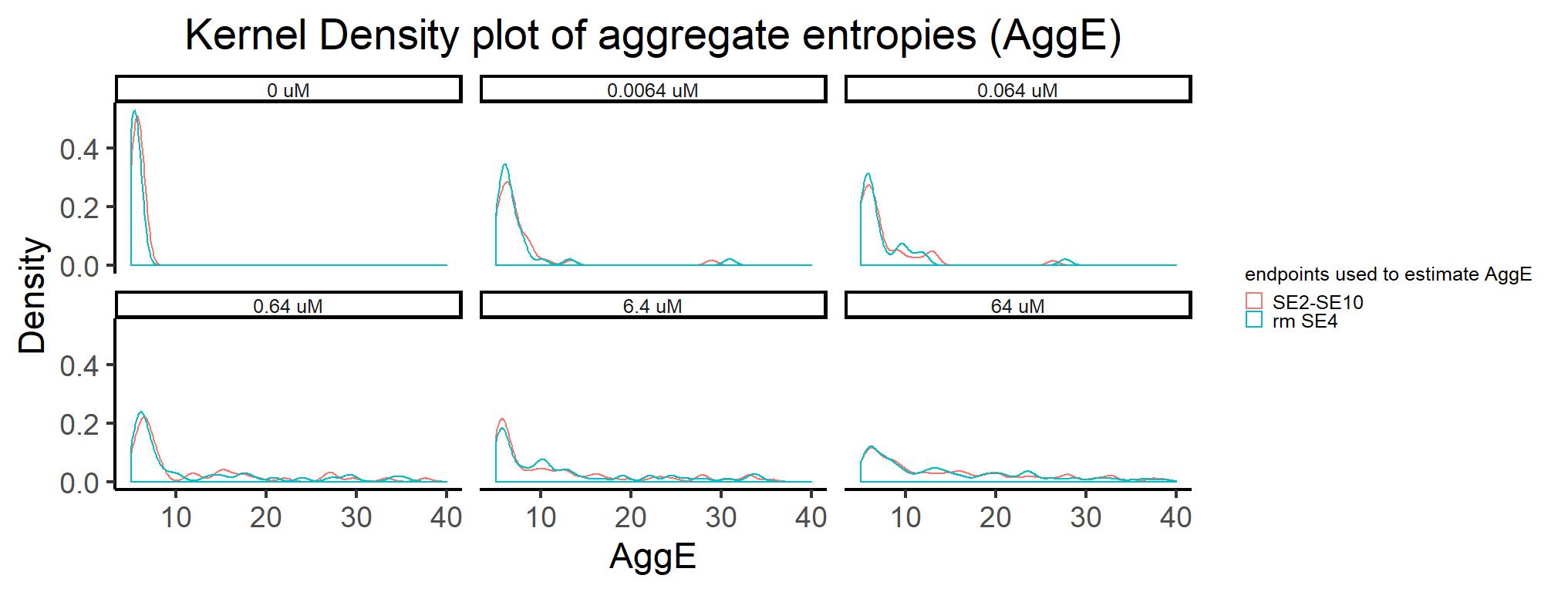


**Supplemental Figure 3** Kernel density plot of Aggregate entropies (AggE) of ToxCast chemicals before and after removing ‘Edema’ (SE4). The density histogram plots AggE on the horizontal axis.


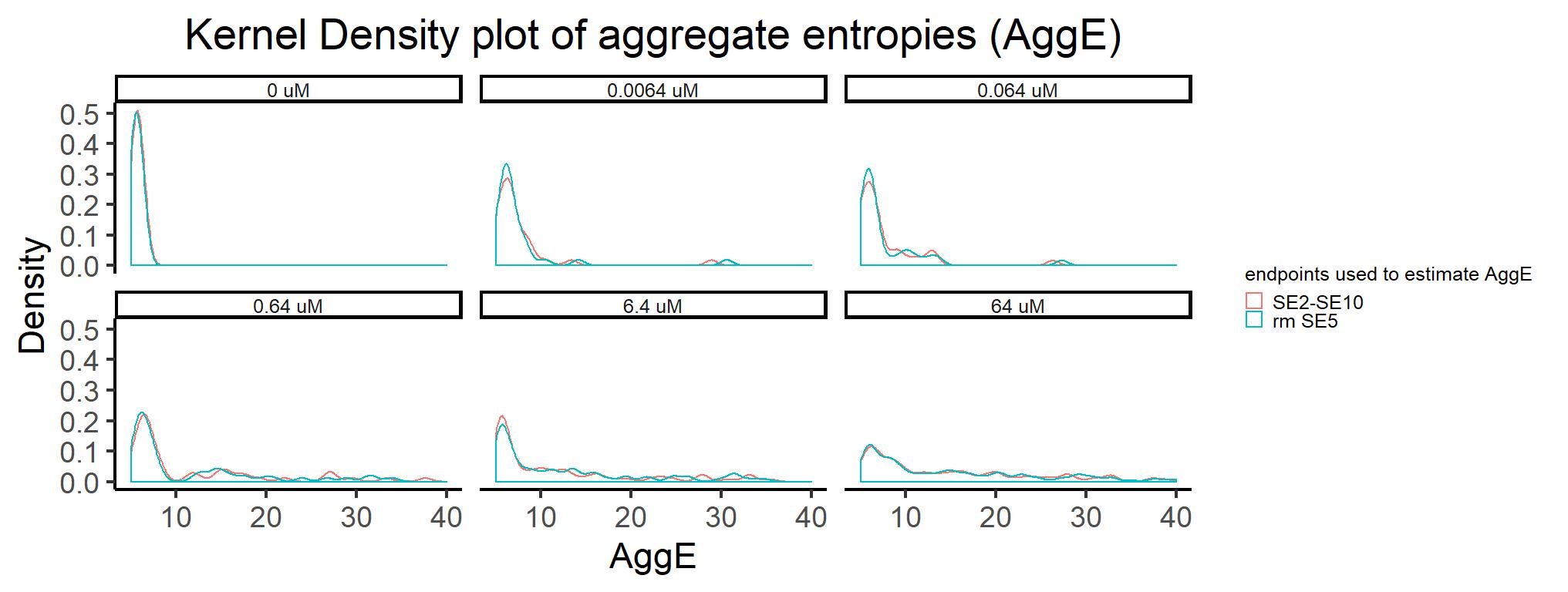


**Supplemental Figure 4** Kernel density plot of Aggregate entropies (AggE) of ToxCast chemicals before and after removing ‘Upright body’ (SE5). The density histogram plots AggE on the horizontal axis.


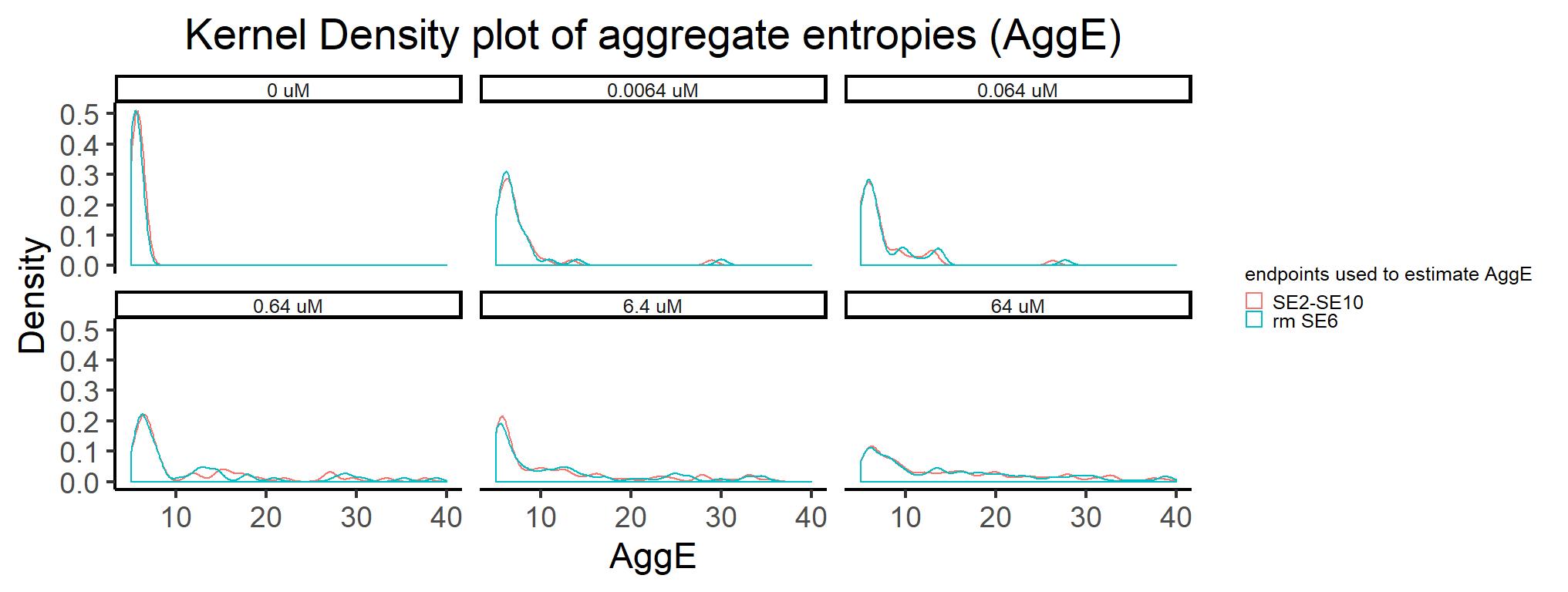


**Supplemental Figure 5** Kernel density plot of Aggregate entropies (AggE) of ToxCast chemicals before and after removing ‘Touch Response’ (SE6). The density histogram plots AggE on the horizontal axis.


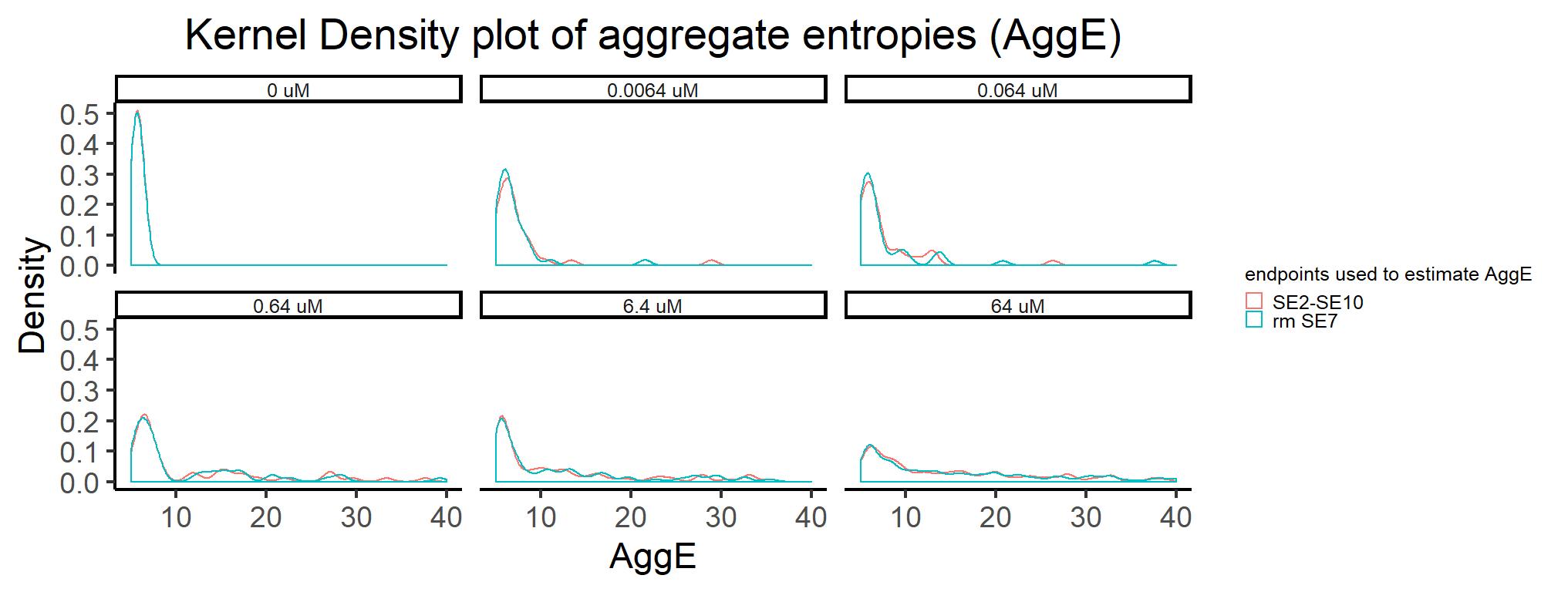


**Supplemental Figure 6** Kernel density plot of Aggregate entropies (AggE) of ToxCast chemicals before and after ‘Pigment’ (SE7). The density histogram plots AggE on the horizontal axis.


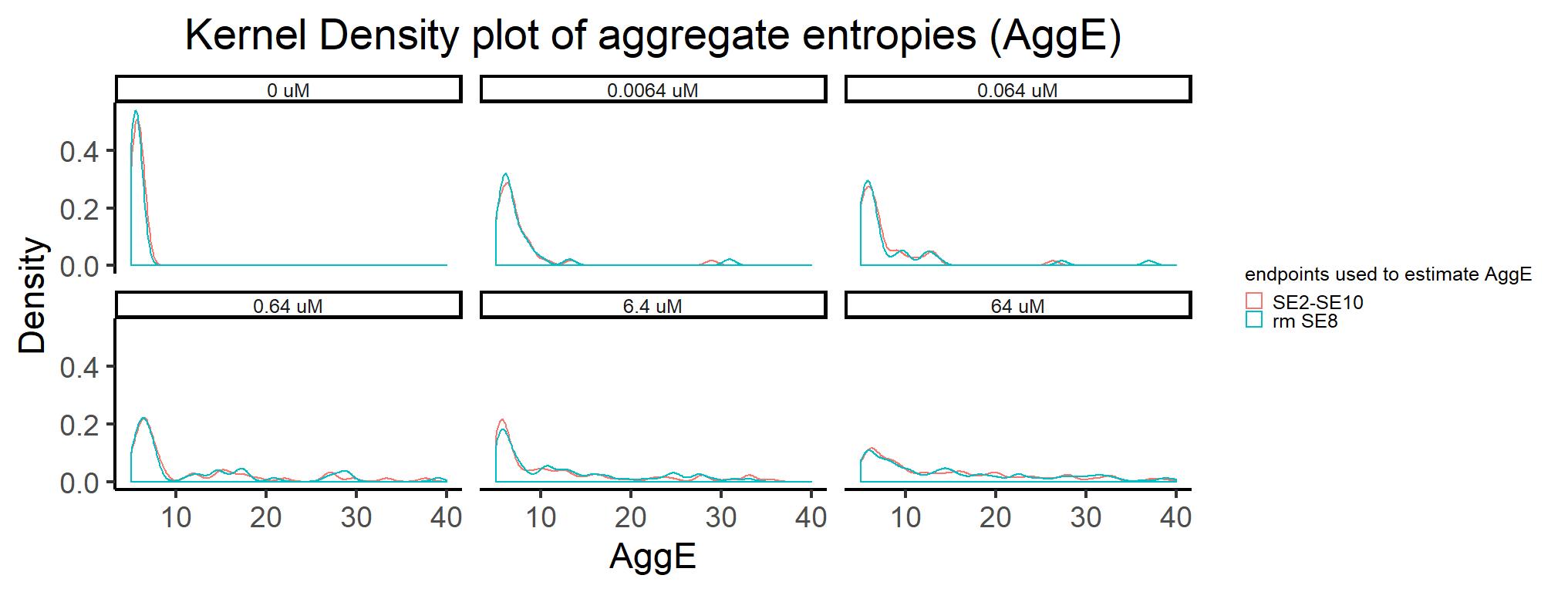


**Supplemental Figure 7** Kernel density plot of Aggregate entropies (AggE) of ToxCast chemicals before and after removing ‘Brain’ (SE8). The density histogram plots AggE on the horizontal axis.


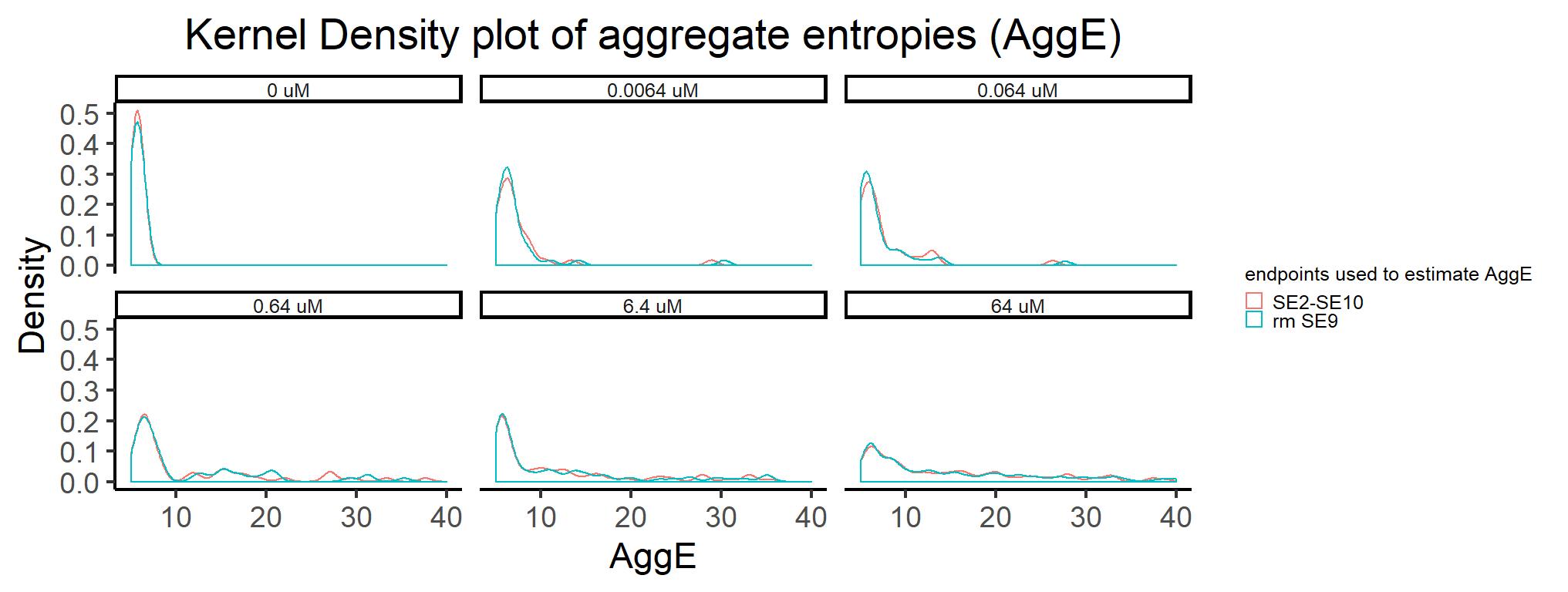


**Supplemental Figure 8** Kernel density plot of Aggregate entropies (AggE) of ToxCast chemicals before and after removing ‘Notochord distortion’ (SE9). The density histogram plots AggE on the horizontal axis.


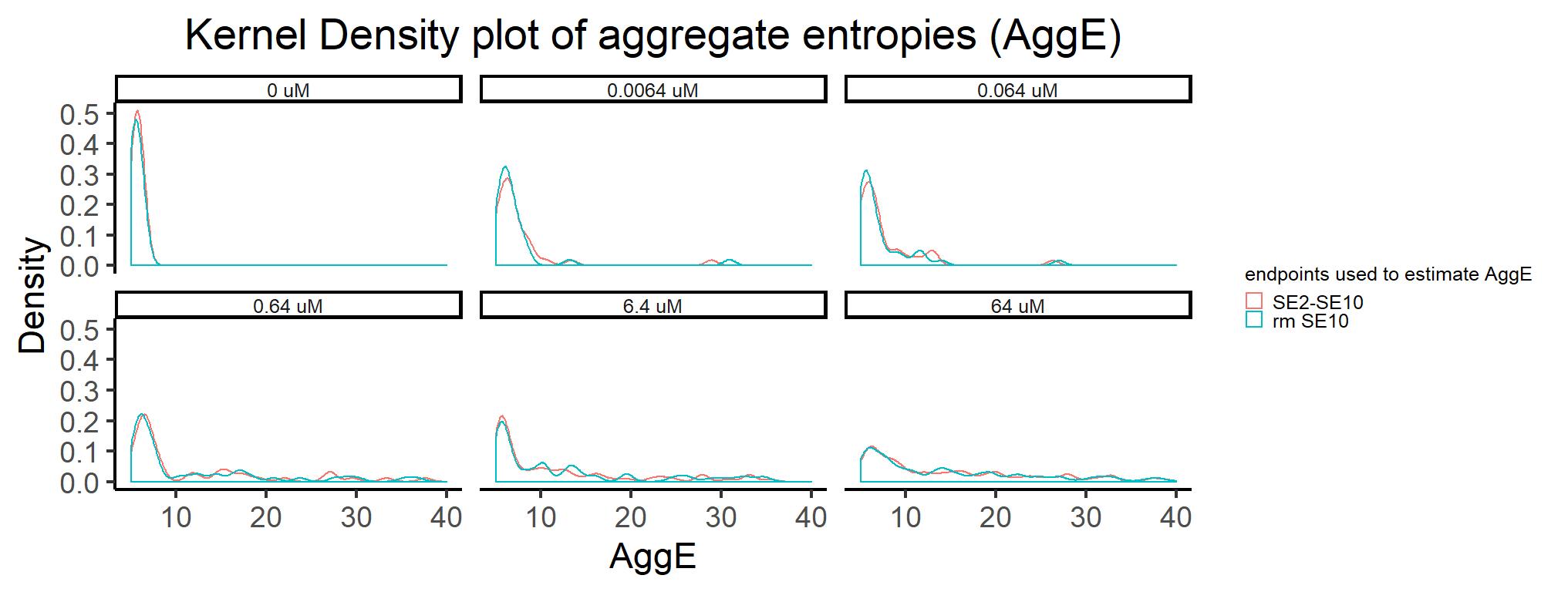


**Supplemental Figure 9** Kernel density plot of Aggregate entropies (AggE) of ToxCast chemicals before and after removing ‘Trunk’ (SE10). The density histogram plots AggE on the horizontal axis.
